# Impact of tailored feedback on optimization and radiation dose reduction in coronary CT angiography: a comparative survey between 2021 and 2023 in Mie prefecture

**DOI:** 10.1007/s11604-025-01835-0

**Published:** 2025-07-19

**Authors:** Suguru Araki, Kakuya Kitagawa, Miyuko Fujita, Shintaro Yamaguchi, Takanori Kokawa, Florian Michallek, Masafumi Takafuji, Satoshi Nakamura, Yasutaka Ichikawa, Hajime Sakuma

**Affiliations:** 1 Department of Radiology, Mie University Hospital, 2-174 Edobashi, Tsu, Mie 514-8507 Japan; 2 Regional Co-Creation Deployment Center, Mie Regional Plan Co-Creation Organization, Mie University, 1557 Kurimamachiyacho, Tsu, Mie 514-8507 Japan; 3 Department of Advanced Diagnostic Imaging, Mie University Graduate School of Medicine, 2-174 Edobashi, Tsu, Mie 514-8507 Japan; 4 Department of Radiology, Charité—Universitätsmedizin Berlin, Charitéplatz 1, 10117 Berlin, Germany

**Keywords:** Coronary computed tomography angiography, Radiation dose, Protocol optimization, Japan, Regional healthcare

## Abstract

**Purpose:**

Despite advances in dose-reduction strategies for coronary CT angiography (CCTA), a 2021 regional survey in Mie Prefecture revealed that the 75th percentile CT dose index volume (CTDIvol) remained 48 mGy—lower than Japan’s 2020 diagnostic reference level (66 mGy), yet substantially exceeding international benchmarks (~ 25 mGy). Tailored feedback based on Society of Cardiovascular Computed Tomography (SCCT) guidelines was disseminated to each institution in 2022. This study aimed to evaluate the impact of these intervention on cardiac CT practice in Mie Prefecture in 2023.

**Materials and methods:**

Institutions with 64-row or greater multidetector CT scanners across Mie Prefecture were invited; 17 hospitals ultimately enrolled. Each site provided CCTA scan protocols and radiation dose data from 20 to 30 consecutive patients aged 20–80 years and weighing 50–70 kg. Examinations performed for coronary artery bypass graft evaluation or aortic valve assessment were excluded. Imaging parameters and radiation dose metrics were compared with a 2021 pre-feedback survey.

**Results:**

Data from 487 patients (median age: 71 years, 62% male) were analyzed. Of the 16 institutions participating in both surveys, 88% (14/16) modified protocols. Prospective ECG-triggered scanning increased (47–68%), retrospective scanning decreased (46–18%), and adoption of low tube potential rose (33–67%). The 75th percentile CTDIvol decreased from 48 to 31 mGy. No increase in adverse events or image quality deterioration was observed; rather, image quality exhibited an upward trend.

**Conclusion:**

Tailored regional feedback substantially improved CCTA radiation practices in Mie Prefecture, achieving significant dose reductions without compromising image quality or requiring equipment upgrades. These findings may inform protocol optimization efforts nationwide.

**Supplementary Information:**

The online version contains supplementary material available at 10.1007/s11604-025-01835-0.

## Introduction

Cardiac computed tomography (CT) has become a cornerstone of non-invasive cardiovascular imaging, offering robust diagnostic and prognostic value in clinical practice [1, 2]. Coronary CT angiography (CCTA) demonstrates a high negative predictive value for ruling out coronary artery disease (CAD) [3] and is associated with a significant reduction in cardiac mortality and non-fatal myocardial infarction [4, 5]. In Japan, the adoption of CCTA continues to expand, driven in part by the 2022 Japanese Cardiovascular Society guidelines endorsing CCTA as a first-line diagnostic modality for chronic CAD [6, 7].

Despite these advances, concerns regarding radiation exposure associated with CCTA persist [8, 9]. According to Japan’s 2020 Diagnostic Reference Levels (DRLs) for cardiac CT—which represent the 75th percentile of median radiation doses reported by surveyed institutions—the reference values for cardiac CT examinations, defined as a dose-length product (DLP) of 1300 mGy·cm and a CT dose index volume (CTDIvol) of 66 mGy [10], remain substantially higher than international benchmarks, including those reported in the PROTECTION VI study (DLP 400 mGy·cm; CTDIvol 24 mGy) [11] and the EUCLID study (DLP 935 mGy·cm; CTDIvol 25 mGy) [12]. This discrepancy reflects both technical factors and variations in clinical practices across Japanese institutions.

A prefecture-wide survey conducted in Mie Prefecture in 2021 revealed that although radiation doses from CCTA examinations were slightly below the national DRLs, they continued to exceed international standards. The survey further identified limited adoption of low tube potential imaging and continued reliance on retrospective ECG-gated scanning as major contributors to elevated radiation exposure [13].

To address these gaps, tailored feedback based on Society of Cardiovascular Computed Tomography (SCCT) guidelines was delivered to participating institutions in 2022. This feedback emphasized heart rate (HR) control, increased use of prospective scanning, and optimization of tube potential selection based on patient characteristics.

The present study evaluates the effectiveness of a regionally tailored feedback program aimed at optimizing cardiac CT practice in Mie Prefecture, as a potential model for broader dose optimization efforts in Japan.

## Materials and methods

### Study protocol

This retrospective observational survey was approved by the Institutional Review Board of Mie University Hospital (approval No. H2023-045), and the requirement for written informed consent was waived because only anonymized data were analyzed. We invited all healthcare institutions in Mie Prefecture equipped with multi-detector row computed tomography (MDCT) scanners with 64 or greater rows to participate in the survey, including private clinics, community hospitals, and Mie University Hospital—the region’s sole academic medical center. As of December 2023, 50 such institutions were identified. The primary survey aimed to determine whether these institutions had performed cardiac CT in 2023 and to record the number of examinations conducted. Responses were obtained from 36 institutions, of which 25 reported performing cardiac CT. These 25 institutions were subsequently invited to participate in a secondary survey, and 22 agreed to cooperate.

In the secondary survey, local collaborators collected image acquisition details and radiation dose reports from 30 consecutive patients who had undergone cardiac CT in clinical practice between January and December 2023. The inclusion criteria were as follows: patients who underwent cardiac CT as part of routine clinical care using MDCT with 64 or more detector rows, aged 20–80 years, and with a body weight of 50–70 kg. This weight range was adopted in the present study to enable direct comparison of radiation dose with previous data, as it was used in the Japan DRLs 2020 and in our 2021 survey. Patients who underwent examinations for post-coronary artery bypass graft evaluation or preoperative assessment before aortic valve surgery were excluded due to the requirement for broader imaging coverage and the heterogeneity of acquisition protocols. Pre-ablation CT scans were included if they were performed simultaneously with coronary artery evaluation. In addition, institutions that had participated in the 2021 survey were asked whether they had implemented any modifications to their protocols based on the tailored feedback previously provided. Image data were not transferred centrally; each site extracted the required information and sent it to the core laboratory using a standardized electronic form.

**Fig. 1:**
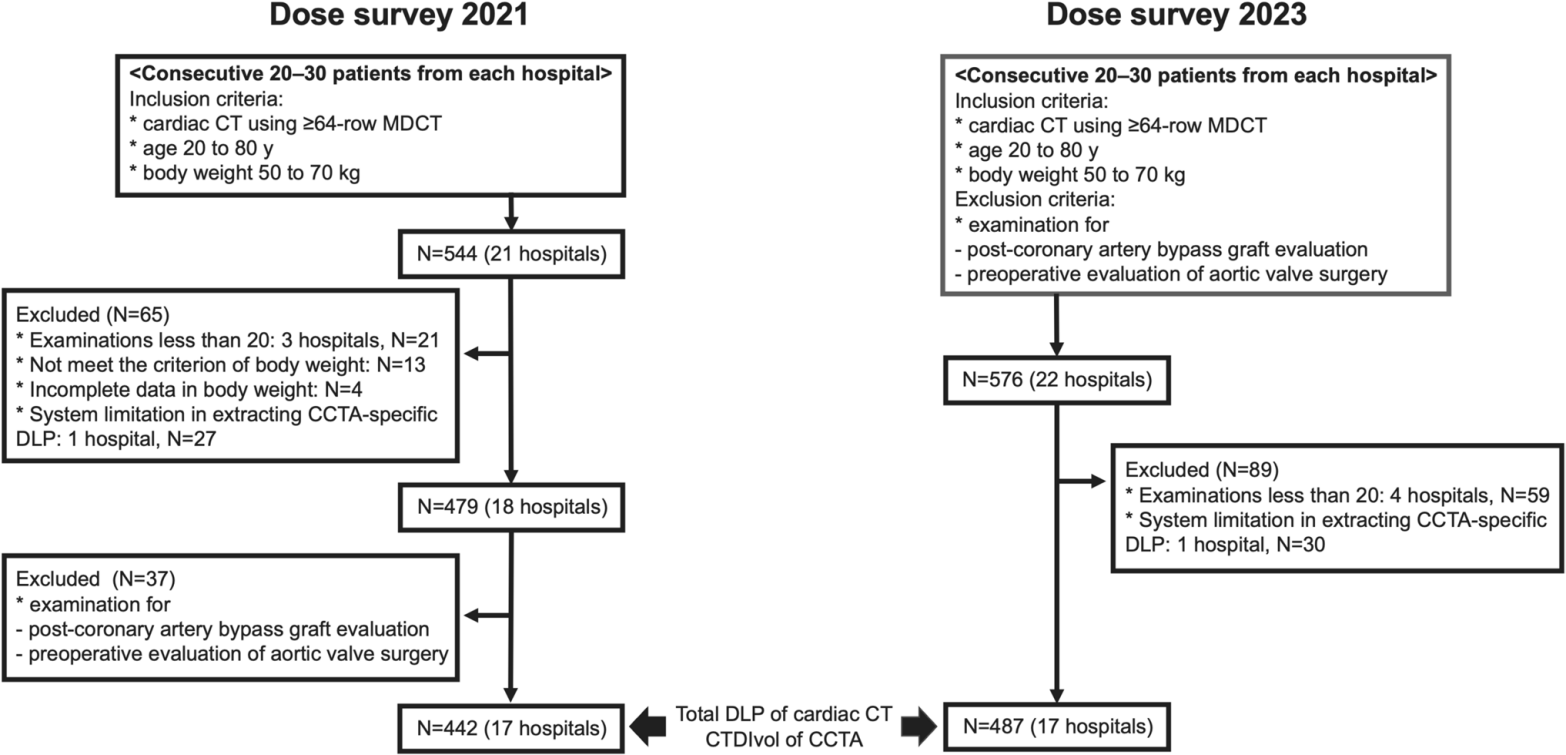
Overview of study design including patient selection protocol in this study. Abbreviations: MDCT, multidetector row computed tomography; DLP, dose-length product; CTDIvol, computed tomography dose index volume; CCTA, coronary computed tomography angiography

In total, data from 576 patients across 22 hospitals were collected. Among them, four hospitals had fewer than 20 eligible patients; therefore, 59 patients from these hospitals were excluded in accordance with the exclusion criteria set by the Japan DRLs 2020 and the previous 2021 dose survey. In addition, one hospital was excluded due to a system limitation that prevented extraction of the DLP specific to the CCTA portion. As a result, a total of 487 patients from 17 hospitals were included in the final analysis. To compare with the previous dose survey conducted in 2021, we retrieved data from 442 patients at 17 hospitals from the previous dose survey [13], using the same inclusion and exclusion criteria as used in the secondary survey of the 2023 dose study (Fig. 1).

All observational data were analyzed at the core laboratory. Patient characteristics collected included: sex, height, body weight, indication for cardiac CT (whether for coronary artery evaluation or other purposes), HR, heart rhythm (stable or non-stable sinus rhythm), and whether nitroglycerin or β-blockers were administered. Imaging parameters recorded included CT system characteristics (number of detector rows and type of detector: energy-integrating detectors [EID] or photon-counting detectors [PCD]), tube potential, image reconstruction method (filtered back projection, iterative reconstruction [IR], or deep-learning iterative reconstruction [DLIR]), and application of automatic exposure control (AEC). The employed scan techniques were also documented, including ECG-gated retrospective helical scanning, ECG-triggered prospective helical scanning, ECG-triggered prospective axial scanning, or Computed Tomography Angiography/Cardiac Function Analysis (CTA/CFA) mode. CTA/CFA is an ECG-gated scan mode of Canon’s 320-row MDCT, which can capture the entire R-R interval without table movement.

**Fig. 2:**
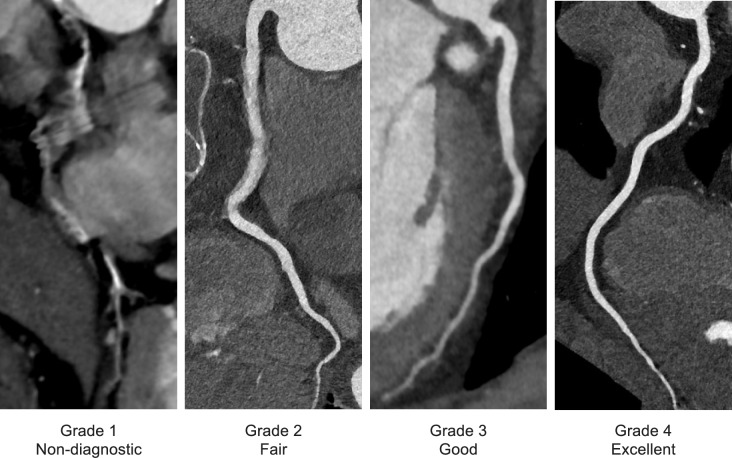
Illustration of image quality scores of coronary arteries on CCTA images. A four-point scale was used: 1, nondiagnostic; 2, fair; 3, good; and 4, excellent. Abbreviation: CCTA, coronary computed tomography angiography

Image quality was assessed by local collaborators at each institution. Observers evaluated the right coronary artery (RCA), left main coronary artery (LMT), left anterior descending artery (LAD), and left circumflex artery (LCX) using a four-point scale, considering factors such as motion artifacts, sharpness, noise, contrast enhancement, and beam hardening, which are crucial for analyzing the morphology of coronary arteries: 1, non-diagnostic (the image quality is so poor that assessing the morphology of coronary arteries and plaque is impossible); 2, fair (the image quality poses some challenges, but morphology assessment is still feasible); 3, good; (the image quality presents minor imperfections, yet allows for morphology assessment without major difficulties) and 4, excellent (the image quality makes assessment straightforward and easy) (Fig. 2) [14, 15]. The average score of the RCA, LMT, LAD, and LCX was used as the image quality score for each patient.

Radiation dose parameters were derived from the dose reports and evaluated based on both the CTDIvol and the DLP for the CCTA portion of the examination.

### Tailored feedback in 2021

Tailored feedback was provided to facilities that participated in the 2021 dose survey. This feedback included an overview of survey results and specific data on cardiac CT radiation doses for each facility. Recommendations for radiation dose reduction were offered, including optimization of tube potential settings and HR control strategies in accordance with SCCT guidelines [16]. Protocol adjustments were also suggested, considering each facility’s CT scanner characteristics, including tube performance, available scan modes, reconstruction methods, and recommendations on scanning range. Examples of the tailored feedback provided to one of the hospitals that participated in the previous survey are presented in Supplementary Fig. 1 in Japanese and Supplementary Fig. 2 with its English translation.

### Statistical analysis

Statistical analysis was carried out using JMP version 14.2.0 (SAS, Institute Cary, NC, USA) software. Continuous data are presented as medians with interquartile ranges (IQR), or as counts with percentages. Comparisons between groups were made using Wilcoxon–Mann–Whitney U test for continuous variables and chi-square tests for categorical variables. For all analyses, statistical significance was defined as p-value < 0.05.

## Results

### Patient and study site characteristics

In the current dose survey, a total of 487 patients were enrolled from 17 hospitals, with a median age of 71 (IQR: 63–75) years and 301 (62%) being men. The median patient height was 162 (IQR: 155–168) cm, weight 61 (IQR: 55–65) kg, and HR at the scan was 59 (IQR: 54–65) beats per minute (bpm). Sinus rhythm was present in 445 (91%) of the patients. In comparison, the previous dose survey included 442 eligible patients from 17 hospitals, with a median age of 70 (IQR: 62–75) years and 269 (61%) being men. The median patient height was 162 (IQR: 156–168) cm, weight 61 (IQR: 55–66) kg, with HR 60 (IQR: 54–66) bpm and sinus rhythm present in 413 (93%) of the patients. Nitroglycerin was administered in 98% and 99% of cases in 2021 and 2023, respectively (p = 0.28). β-blockers were used in 63% of patients in 2021 and 61% in 2023 (p = 0.46) (Table 1).

**Table 1:** Patient characteristics

	Dose survey 2021	Dose survey 2023	p value
	N = 442	N = 487	
Age [y]	70 (62–75)	71 (63–75)	0.15
Sex, men	269 (61%)	301 (62%)	0.77
Height [cm]	162 (156–168)	162 (155–168)	0.82
Weight [kg]	61 (55–66)	61 (55–65)	0.85
Heart rate [beats/min]	60 (54–66)	59 (54–65)	0.25
Heart rhythm			0.24
Sinus rhythm	413 (93%)	445 (91%)	
Others	29 (7%)	42 (9%)	
Nitroglycerin	435 (98%)	483 (99%)	0.28
Beta-blocker	279 (63%)	296 (61%)	0.46

Data are presented as the median (interquartile range) or number of patients (%)

**Fig. 3:**
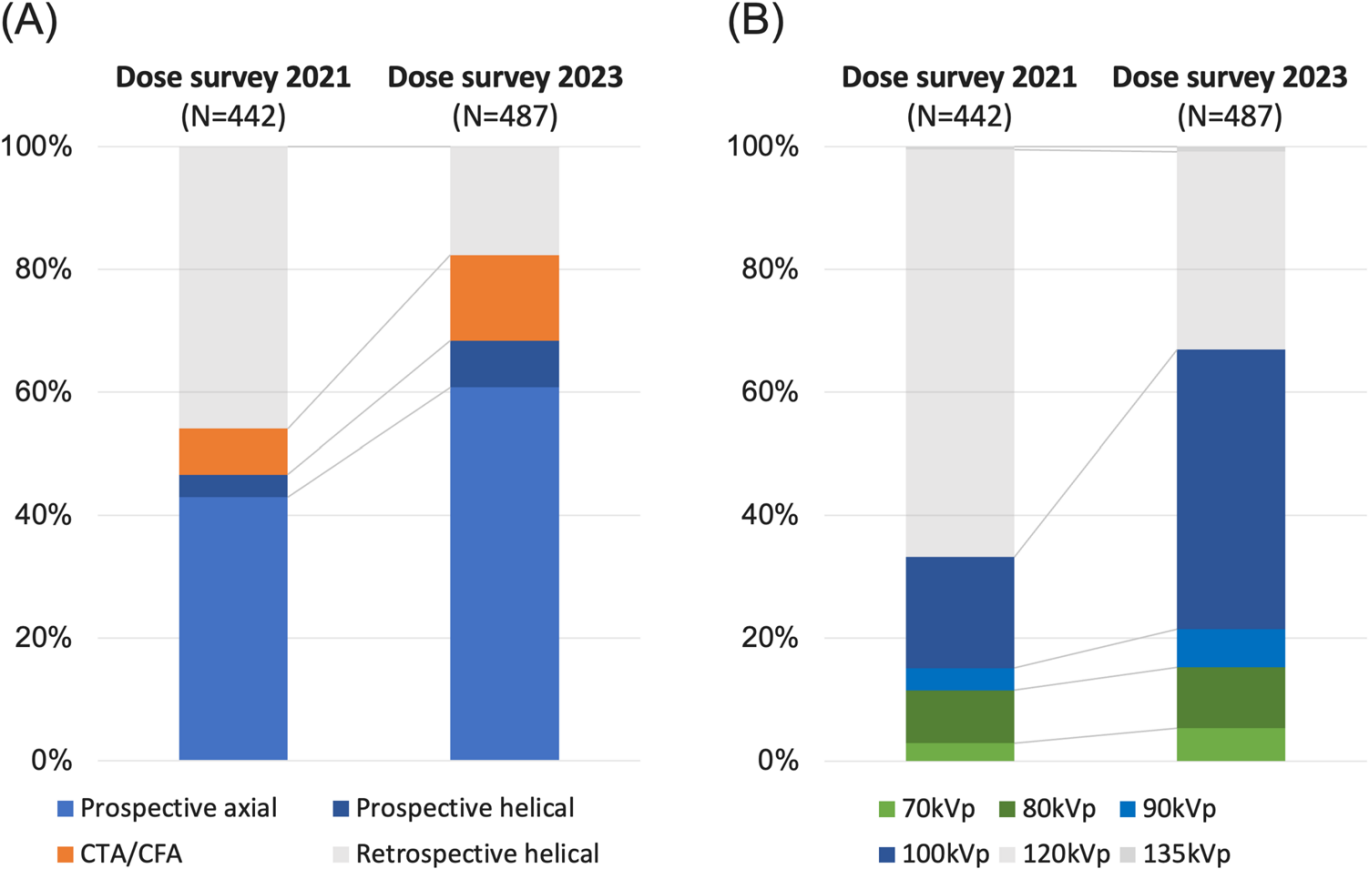
Scan techniques and tube potentials used in the previous and current dose surveys. **A** Proportions of scan techniques in the 2021 and 2023 surveys. **B** Proportions of tube potentials in the 2021 and 2023 surveys. Abbreviations: CTA/CFA, Computed Tomography Angiography/Cardiac Function Analysis

In the current dose survey, 252 (52%) of scans were performed using area-detector CT scanners, 118 (24%) using dual-source CT scanners with EID, and 8 (2%) using dual-source CT scanners with PCD. In contrast, in the previous dose survey, 187 (42%) of scans were performed using area-detector CT scanners and 108 (25%) using dual-source CT scanners with EID. The characteristics of the CT scanners used in the dose survey 2021 and 2023 are listed in Supplementary Table 1. AEC were used in almost all scans for both the current and previous dose surveys. Regarding image reconstruction techniques, no cases used filtered back projection. All scans were reconstructed using either conventional IR or DLIR. In the 2023 survey, IR and DLIR were used in 346 (71%) and 141 (29%) cases, respectively, whereas in the 2021 survey, the numbers were 366 (83%) and 76 (17%), respectively. Regarding scan techniques, ECG-gated retrospective helical scanning accounted for 18% of all scans in the current survey, down from 46% in the previous survey. In contrast, the use of prospective ECG-triggered scanning increased from 47 to 68%. Low tube potential scanning was employed in 67% of the scans in the current survey, whereas it was used in 33% of scans in the previous survey (Table 2, Fig. 3).

**Table 2:** Imaging characteristics

	Dose survey 2021	Dose survey 2023	p value
	N = 442	N = 487	
CT characteristics			< 0.01
Area (16 cm or 8 cm) -detector CT	187 (42%)	252 (52%)	
Standard CT (single source, −4 cm)	147 (33%)	118 (24%)	
Dual-source CT (EID)	108 (25%)	109 (22%)	
Dual-source CT (PCD)	0 (0%)	8 (2%)	
CT manufacturer			< 0.01
Canon	230 (52%)	258 (53%)	
Siemens	138 (31%)	170 (35%)	
Philips	55 (13%)	30 (6%)	
GE	19 (4%)	29 (6%)	
Image reconstruction technique			< 0.01
Conventional IR	366 (83%)	346 (71%)	
Deep-learning IR (DLIR)	76 (17%)	141 (29%)	
Automatic Exposure Control	440 (99.5%)	487 (100%)	0.44
Scan technique			< 0.01
ECG-gated retrospective helical	203 (46%)	86 (18%)	
ECG-triggered prospective helical	16 (4%)	37 (7%)	
ECG-triggered prospective axial	190 (43%)	296 (61%)	
CTA/CFA	33 (7%)	68 (14%)	
Tube potential			< 0.01
135 kVp	2 (1%)	4 (1%)	
120 kVp	293 (66%)	156 (32%)	
90, 100 kVp	96 (22%)	250 (52%)	
70, 80 kVp	51 (11%)	74 (15%)	

Data are presented as number of patients (%)

*CT* computed tomography, *EID* energy integrated detector, *PCD* photon counting detector, *ECG* electrocardiogram, *CTA/CFA* continuous computed tomography angiography/cardiac function analysis

### Association between heart rate distribution and trends in scan techniques

In the previous survey, the HR during scanning was below 60 bpm in 212 patients, between 60 and 64 bpm in 109 patients, and 65 bpm or above in 121 patients. In the current survey, 256 patients had HR below 60 bpm, 116 had HR between 60 and 64 bpm, and 115 had HR 65 bpm or above. The proportion of patients with HR below 65 bpm was 73% in the previous survey, compared to 76% in the current survey (p = 0.31).

**Fig. 4:**
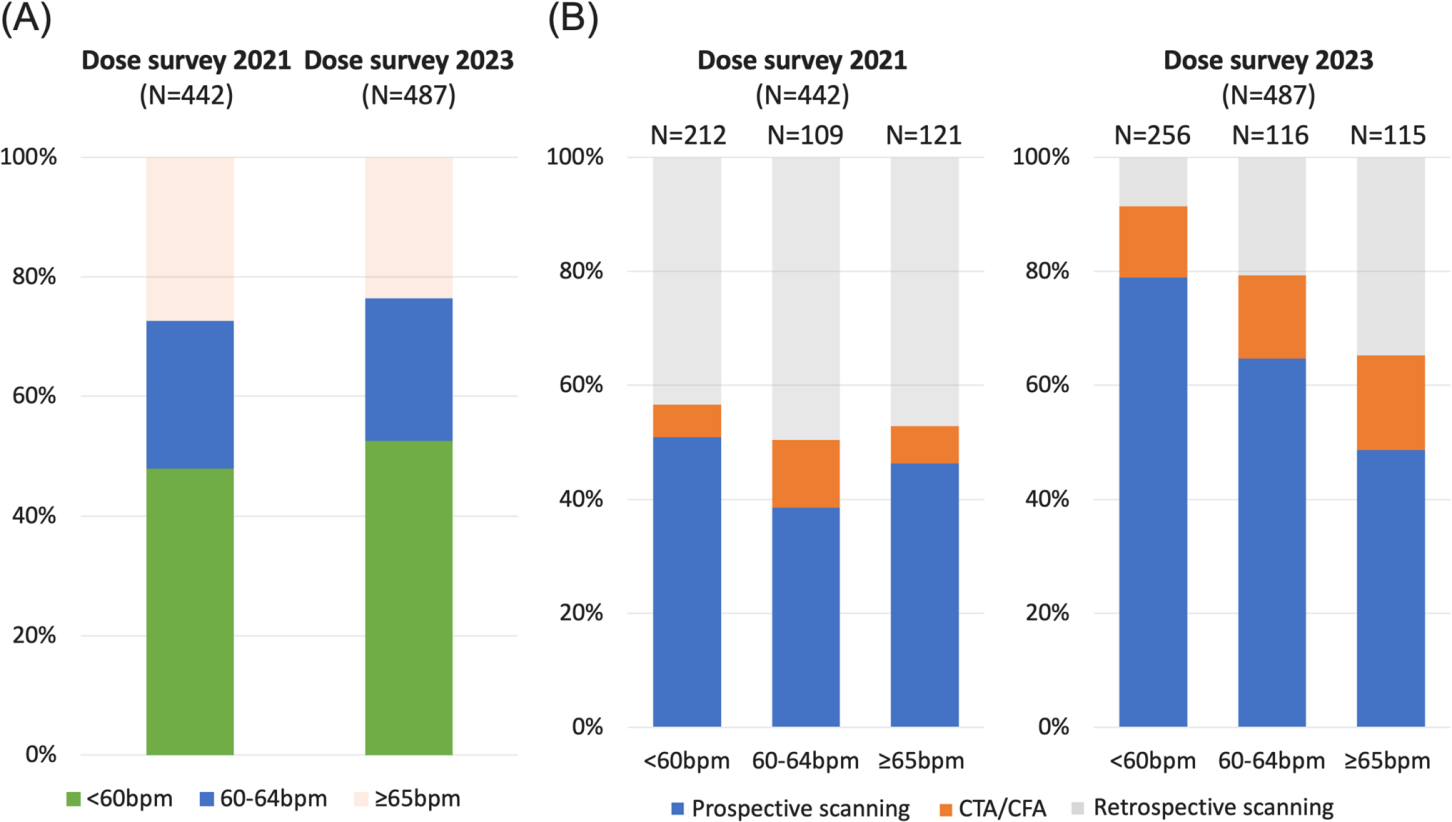
Heart rate during scanning and scan techniques by heart rate in the previous and current dose surveys. **A** Proportions of patients classified by heart rate categories (< 60 bpm, 61–64 bpm, and ≥ 65 bpm) in the 2021 and 2023 surveys. **B** Proportions of scan techniques used within each heart rate category in the 2021 and 2023 surveys. Abbreviations: bpm, beats per minute; CTA/CFA, Computed Tomography Angiography/Cardiac Function Analysis

Regarding scan techniques, in the previous survey, approximately 50% of the scans were performed by using ECG-gated retrospective scanning, regardless of HR (43% for HR below 60 bpm, 50% for HR 60–64 bpm, and 47% for HR 65 bpm or above). In the current survey, the use of ECG-gated retrospective scanning significantly decreased, particularly in patients with lower HR: 9% for HR below 60 bpm, 21% for HR 60–64 bpm, and 35% for HR 65 bpm or above (Fig. 4).

### Image quality of coronary CT angiography

The image quality scores of CCTA were a median of 3.5 (IQR: 3–4) in the previous survey and a median of 3.75 (IQR: 3.25–4) in the current survey, respectively (p < 0.01). In the current dose survey, according to HR categories, the median scores were 3.75 (IQR: 3.5–4) for HR below 60 bpm, 3.75 (IQR: 3.25–4) for HR 60–64 bpm, and 3.5 (IQR: 3–4) for HR 65 bpm or above (p < 0.01). When categorized by tube potential, the image quality scores were 3.5 (IQR: 3–4) for 120 kVp or above and 3.75 (IQR: 3.5–4) for 100 kVp or below (p < 0.01), respectively. In the current survey, image quality was also significantly higher in the DLIR group than in the IR group, with median scores of 4 (IQR: 3.5–4) and 3.75 (IQR: 3–4), respectively (p < 0.01).

### Review of cardiac CT examination protocols of each institution

Sixteen institutions provided valid data for both secondary surveys, having not been excluded based on the criterion of submitting fewer than 20 cases. Among them, two institutions had changed equipment since the previous survey. Cardiac CT protocols were modified 14 (88%) of the institutions. Six institutions adjusted the HR threshold for administering oral or intravenous β-blockers, six decreased tube potential for non-obese patients, and six modified the scanning mode dependent on HR and rhythm. Additionally, four hospitals changed the scanning range, and one adjusted the image noise settings. No complaints, including degradation of image quality or workflow issues, were reported following the protocol modification. However, four institutions continued to use standard tube potential imaging at 120 kVp for over 80% of cases. The reasons for employing 120 kVp included concerns about image degradation at lower tube voltages due to relatively older CT systems in three hospitals, and the need to reduce noise associated with the routine use of sharp kernels which is preferred by the attending radiologist in one.

### Total DLP from cardiac CT and CTDIvol of coronary CT angiography

**Fig. 5:**
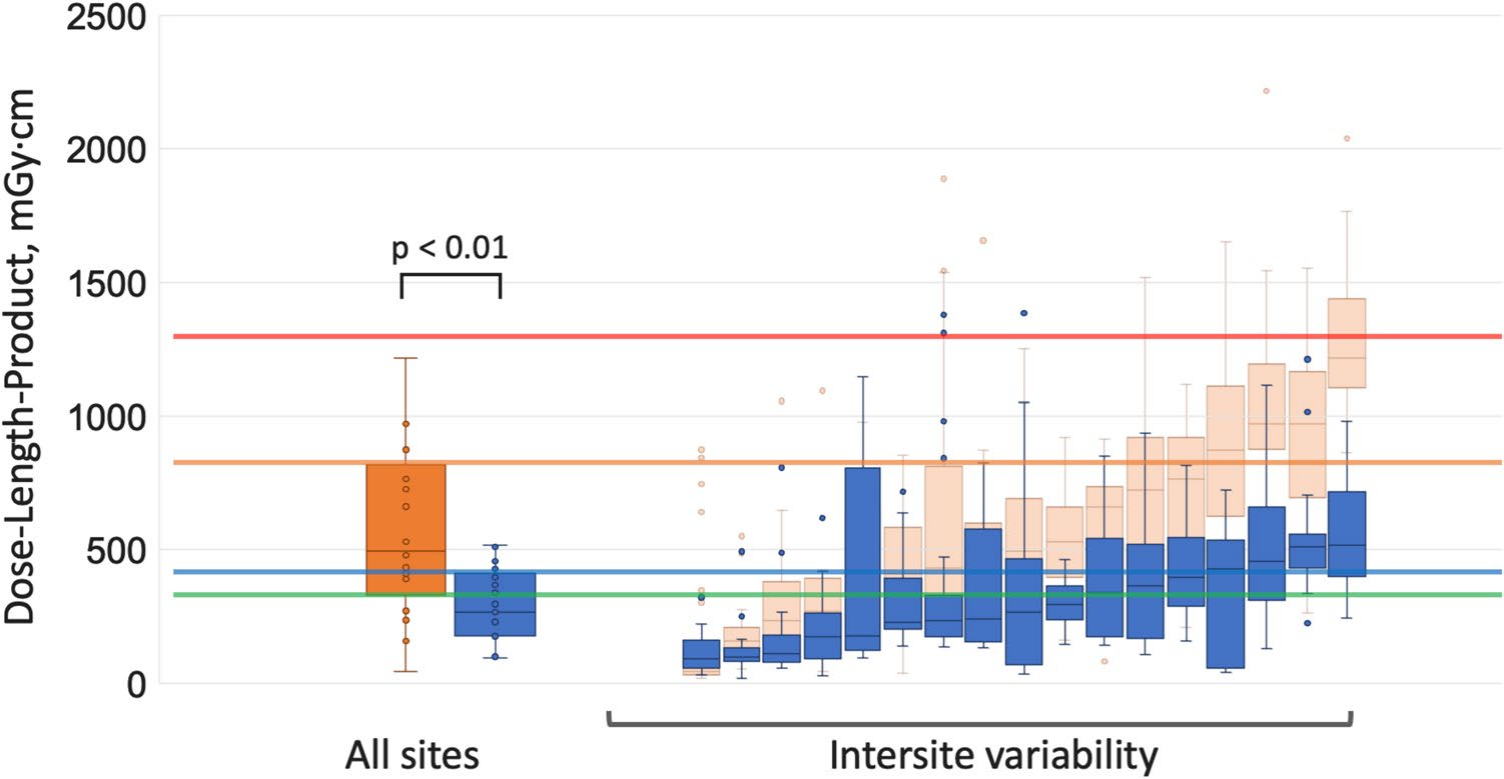
Dose-length product for coronary computed tomography angiography across sites in the previous and current surveys. Left: Box plots showing the DLP for all CCTA examinations. Right: Variability of median DLP (± IQR) among sites. Reference lines: red, Japan DRLs 2020 (1300 mGy·cm); orange, previous survey (764 mGy·cm); blue, current survey (395 mGy·cm); green, PROTECTION VI study (338 mGy·cm). Abbreviations: DLP, dose-length product; CT, computed tomography; DRL, diagnostic reference level

**Fig. 6:**
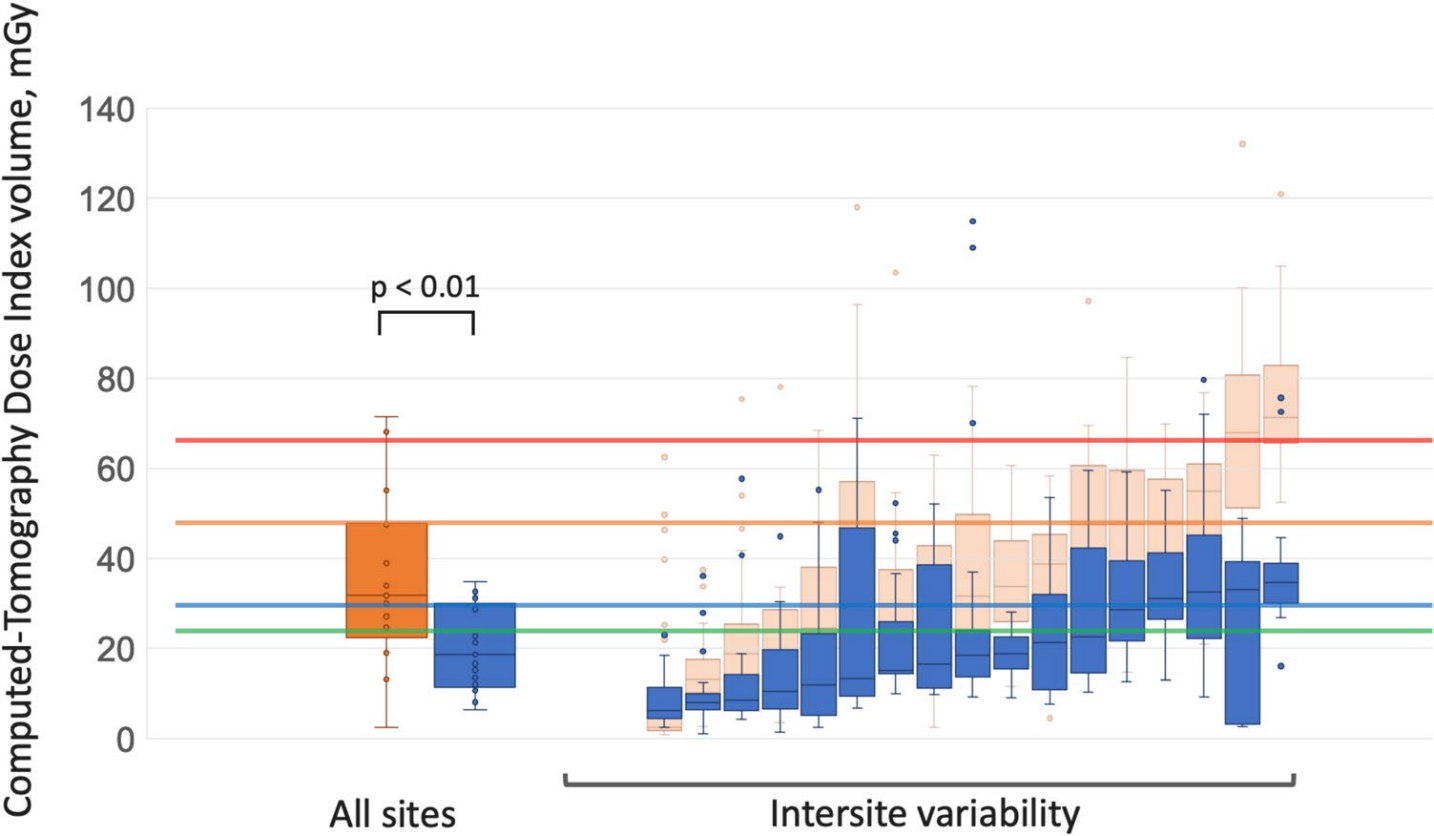
Computed tomography dose index volume for coronary computed tomography angiography across sites in the previous and current surveys. Left: Box plots showing the CTDIvol for all CCTA examinations. Right: Variability of median CTDIvol (± IQR) among sites. Reference lines: red, Japan DRLs 2020 (66 mGy); orange, previous survey (48 mGy); blue, current survey (31 mGy); green, PROTECTION VI study (24 mGy). Abbreviations: CTDIvol, computed tomography dose index volume; CT, computed tomography; DRL, diagnostic reference level

The DLP and CTDIvol for the CCTA portion at each hospital are shown in Fig. 5 and Fig. 6, respectively. In the present study, the median DLP and CTDIvol for CCTA were 266 (176–395) mGy·cm and 19 (12–31) mGy, respectively. Both values were significantly lower than those in the previous survey, where the median DLP was 494 (390–764) mGy·cm and the CTDIvol was 33 (25–48) mGy (p < 0.01 for both), approaching the levels reported in the PROTECTION VI study. Supplementary Table 2 summarizes the DLP and CTDIvol for CCTA in both the previous and current surveys, along with the annual number of cardiac CT examinations performed and the number of hospital beds at each participating facility.

The relationship between scan techniques and CTDIvol for CCTA in the 2021 and 2023 surveys is shown in supplementary Fig. 3. The CTDIvol for CCTA using ECG-triggered prospective scanning, CTA/CFA mode, and ECG-gated retrospective scanning were 19.7 mGy (10.8–34.1), 30.7 mGy (22.4–37.7), and 49.4 mGy (37.6–63.4) in 2021, and 14.4 mGy (9.2–22.5), 27.3 mGy (10.8–36.5), and 39.5 mGy (30.2–45.6) in 2023, respectively.

## Discussion

The present study demonstrates that tailored institutional feedback can meaningfully promote radiation dose reduction in CCTA across a regional healthcare network. The interventions facilitated greater adoption of dose-saving strategies—including the use of low tube potential and prospective ECG-triggered acquisition—particularly among patients with lower HRs, without compromising image quality or generating workflow-related concerns.

Approximately one year after the dissemination of tailored feedback, a follow-up dose survey revealed a 40% reduction in radiation exposure during CCTA examinations, achieved primarily through standardized protocol optimization aligned with SCCT guidelines. Notably, these improvements were realized without substantial equipment upgrades, relying predominantly on optimized scan protocols, guided by individualized feedback adapted to each institution’s scanner capabilities.

The observed reductions in CTDIvol and DLP align with findings from previous large-scale initiatives. For example, Raff et al. reported a 53.3% (from 21 to 10 mSv) reduction in median radiation dose following a statewide educational program that emphasized HR control and scan range limitation, although prospective ECG-triggered techniques were not widely adopted at that time [17]. Similarly, Hamilton-Craig et al. demonstrated that comprehensive education and protocol standardization at a tertiary center yielded a 67% reduction in radiation dose (from 8.4 to 2.8 mSv), highlighting the importance of protocol optimization over hardware replacement [18]. Our study extends these findings by demonstrating that significant radiation dose reductions can be achieved through customized, practical feedback.

While individualized feedback was effective in this regional initiative—where the number of hospitals was limited and many were influenced by the local university hospital—implementing the same approach nationwide would be challenging, particularly in large urban areas like Tokyo or Osaka, where healthcare systems are more fragmented. Cardiac CT inherently requires patient-specific protocol decisions, making standardized practice difficult. Nevertheless, in many institutions, physicians and technologists lack sufficient training in scan optimization. Therefore, establishing a standardized educational framework and formal certification system may offer a more practical and sustainable solution. International models— such as the Certification Board of Cardiovascular Computed Tomography (CBCCT) and Cardiac CT certificate of Advanced Proficiency (CoAP) in the United States, and the European Association of Cardiovascular Imaging (EACVI) Certification and European Board of Cardiovascular Radiology (EBCR) Diploma in Europe—provide structured pathways to ensure practitioner competence. A similar certification structure in Japan, supported by appropriate incentives, could promote consistent dose optimization and overall quality improvement in cardiac CT practice.

Importantly, institutions that modified their protocols reported no increase in adverse events associated with the use of HR control medications, such as β-blockers. Compared to the previous survey, image quality was not degraded—and in fact showed a slight improvement. The exact reason for this trend is unclear, but possible contributing factors include changes in reconstruction methods, such as increased use of DLIR, as well as differences in participating institutions or CT systems between the two surveys.

This study has several limitations. Its retrospective design and reliance on local collaborators for patient selection and image quality evaluation may introduce selection and reporting biases. Additionally, the relatively short interval between feedback intervention and follow-up survey may not fully capture the long-term sustainability of practice changes.

Nonetheless, the findings of this study support the potential of regionally tailored feedback drive meaningful improvements in clinical practice, reduce radiation exposure, and elevate local standards toward alignment with international benchmarks, without necessitating major technological investments. These findings may also serve as a useful reference for protocol refinement in other regions within Japan or in countries facing similar challenges in radiation optimization.

## Supplementary Information

Below is the link to the electronic supplementary material.

Supplementary file1 (XLSX 11 KB)

Supplementary file2 (XLSX 14 KB)

Supplementary file3 (PDF 114 KB)

Supplementary file4 (PDF 646 KB)

Supplementary file5 (PDF 629 KB)
