# Supplementary material for: Impact of tailored feedback on optimization and radiation dose reduction in coronary CT angiography: a comparative survey between 2021 and 2023 in Mie prefecture
Source: Jpn J Radiol. 2025 Jul 19;43(11):1833–41. doi: 10.1007/s11604-025-01835-0 (PMC12575519; doi:10.1007/s11604-025-01835-0)
Supplement: Supplementary file 3 — Supplementary file3 (PDF 114 KB) [file 11604_2025_1835_MOESM3_ESM.pdf]

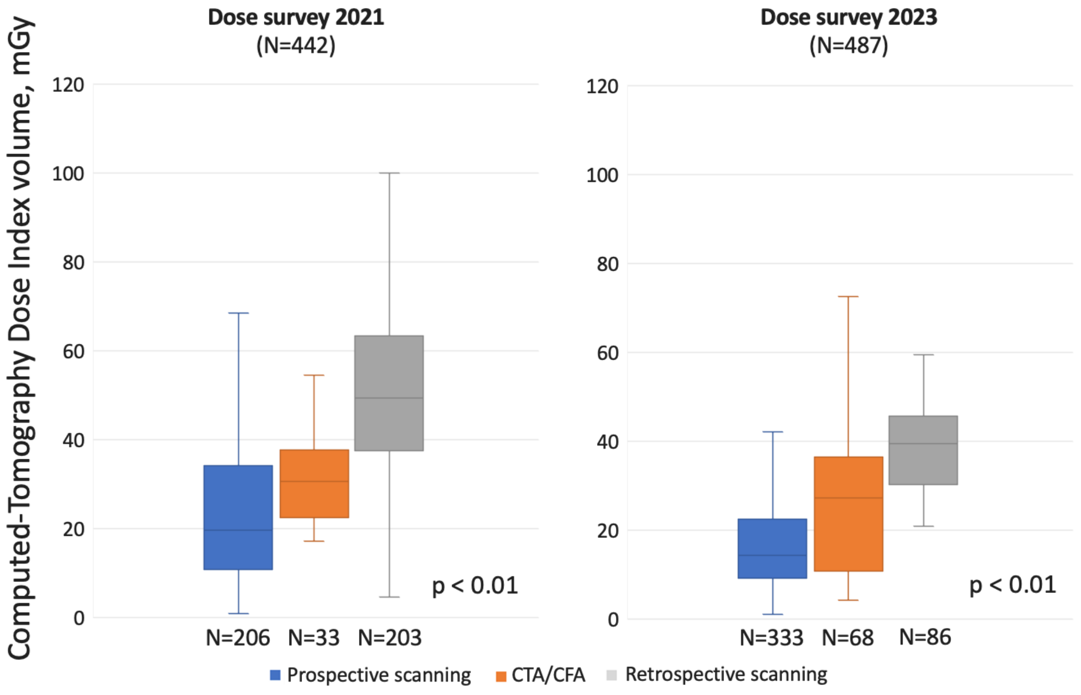

CTDIvol for different CCTA scan techniques in the previous and current surveys.

Median CTDIvol (± IQR) for ECG-triggered prospective scanning, CTA/CFA mode, and ECG-gated retrospective scanning in the 2021 and 2023 surveys.

Abbreviations: CTA/CFA, Computed Tomography Angiography/Cardiac Function Analysis
