# Supplementary material for: Impact of tailored feedback on optimization and radiation dose reduction in coronary CT angiography: a comparative survey between 2021 and 2023 in Mie prefecture
Source: Jpn J Radiol. 2025 Jul 19;43(11):1833–41. doi: 10.1007/s11604-025-01835-0 (PMC12575519; doi:10.1007/s11604-025-01835-0)
Supplement: Supplementary file 4 — Supplementary file4 (PDF 646 KB) [file 11604_2025_1835_MOESM4_ESM.pdf]

三重県における心臓 CT 線量調査（2021 年）

~〇〇〇病院~

【Figure1. 心臓 CT 検査の総 DLP(mGy・cm)】

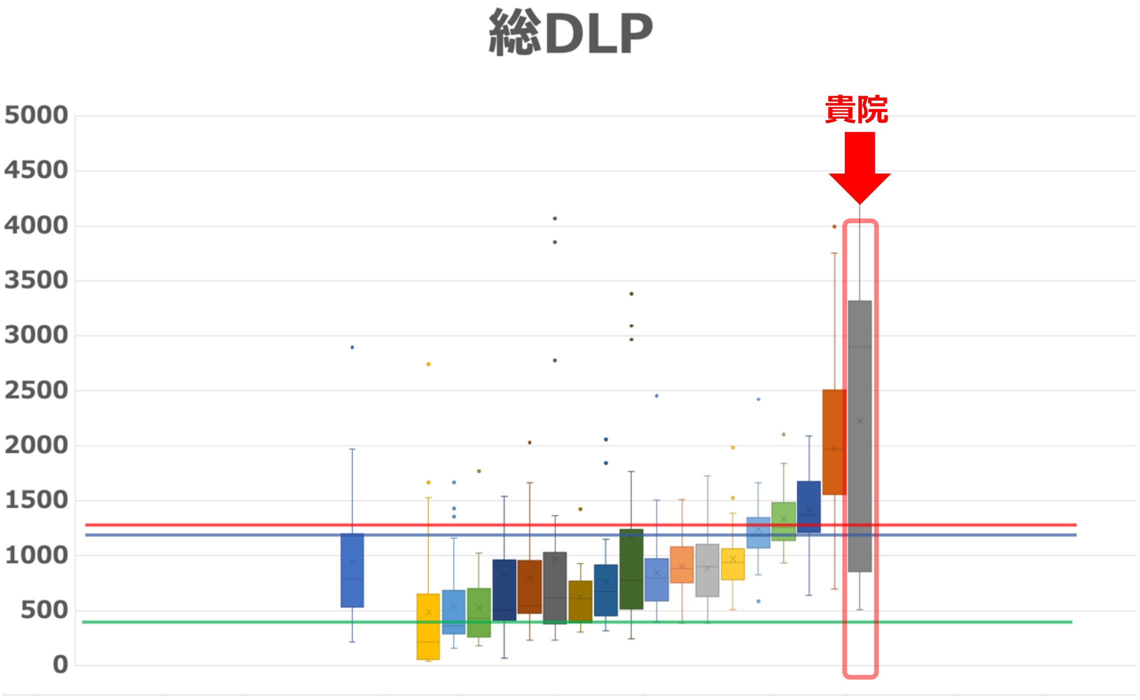

【Figure 2. 心臓 CT 検査の冠動脈 CT angiography (CCTA)部分の CTDIvol (mGy)】

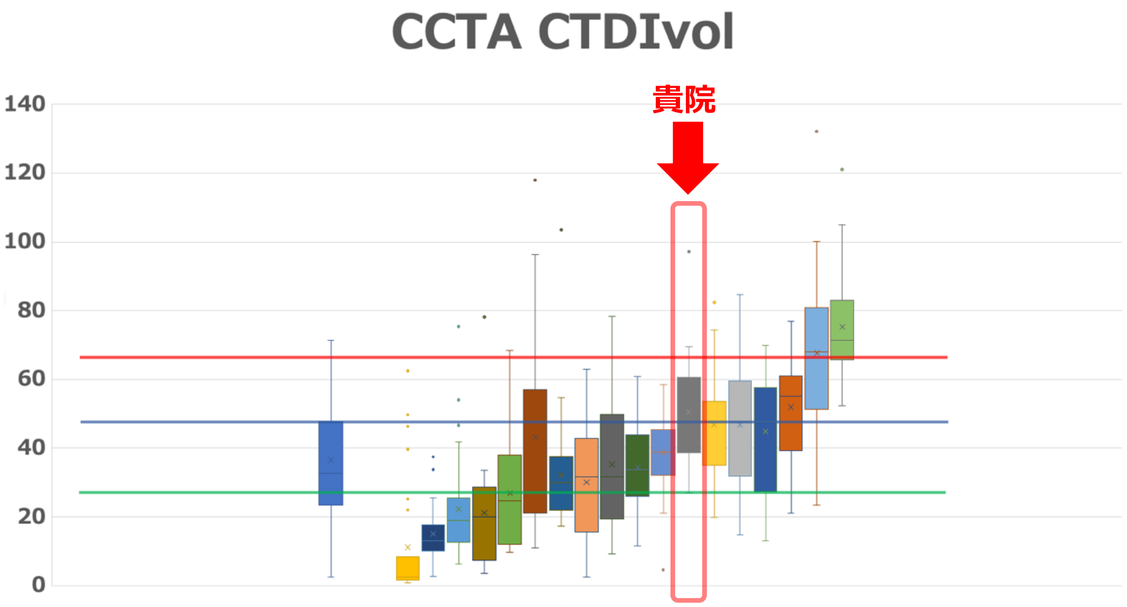

*赤線: Japan DRLs 2020 の DRL　　青線: 三重県(今回の調査)の DRL

緑線: PROTECTION VI（2017 年、世界 61 施設が参加）の DRL

【Figure 3. 撮影方法の内訳（順は Figure 2 と同じ）】

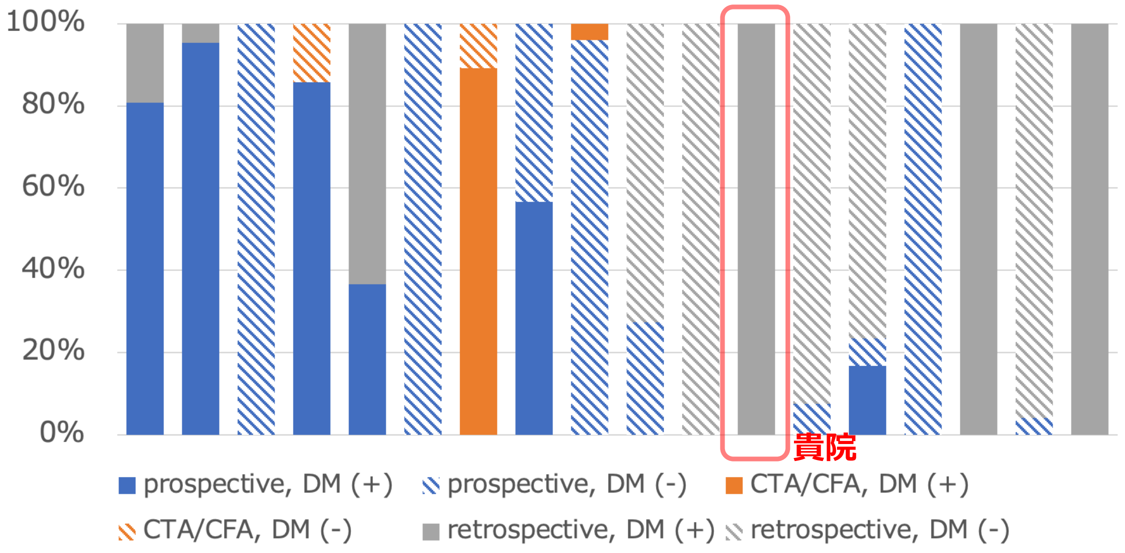

*DM: dose modulation (ECG controlled dose pulsing)
CTA/CFA: Aquilion One に搭載されている撮影モード

【Figure 4. 撮影時の心拍数コントロール状況（順は Figure 2 と同じ）】

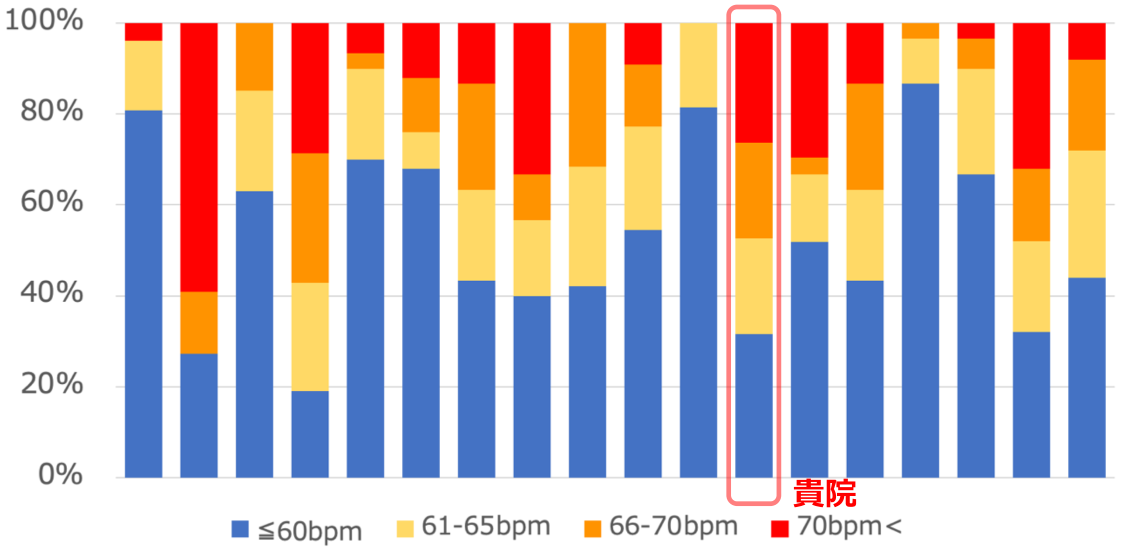

【Figure 5. 管電圧(順は Figure 2 と同じ)】

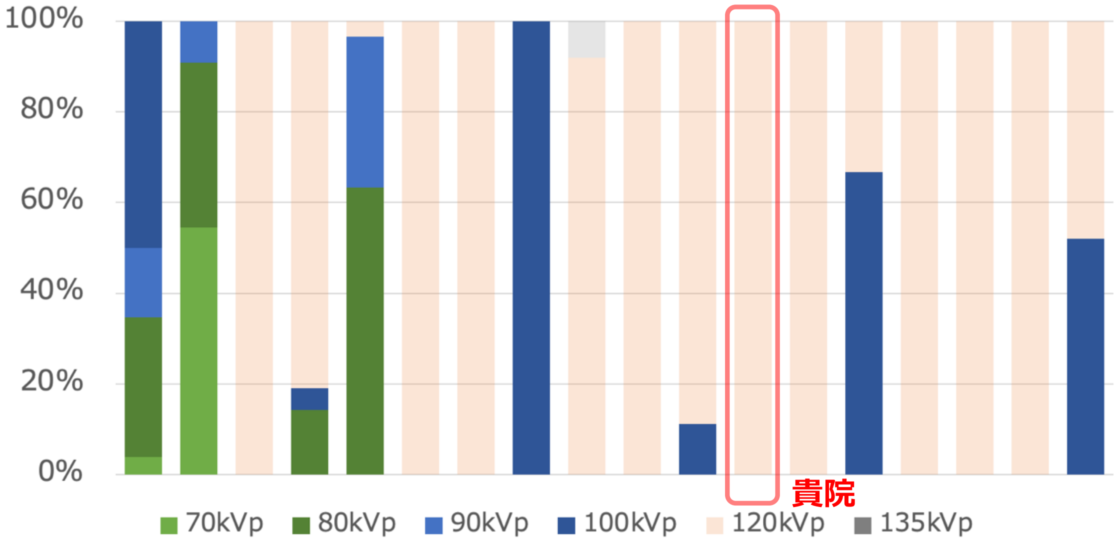

【Figure 6. CCTA の線量に関係する因子】

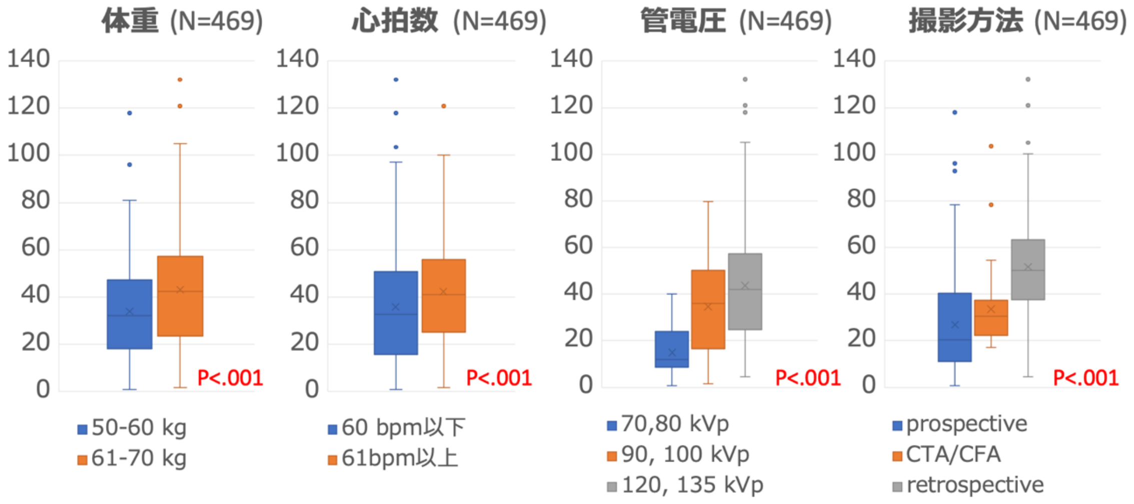

【県内施設と比較した貴院の心臓 CT 検査の現状】

・冠動脈 CT の被曝量は Japan DRLs 2020 と比較すると妥当な線量ですが、県内ではわずかに被曝線量が多い。海外の施設と比較すると高い値になっています。
・心臓 CT 検査全体の被曝量としては県内施設や Japan DRLs 2020 と比較しても高くなっています。「冠動脈 CT 検査」としては余剰な撮影が多い可能性があります。
・心拍数コントロールは改善の余地があります。
・低管電圧撮影は行われていません。
・prospective scan が活用されていません。

【被ばく線量さらなる低減のために】

・低管電圧撮影の活用により、さらなる被曝低減が見込めます。(SCCT のガイドラインでも体重 100kg 以下ないし BMI 30 以下では 100kVp を、小児や小柄な成人では 100kVp または 80kVp が推奨されています。(Abbara S, et al. J Cardiovasc Comput Tomogr 2016; 10: 435-449.)

・施設で既に心拍数コントロールの基準は定めていただいていますが、カットオフの厳格化は考慮しても良いかもしれません。(例えば、「HR 60bpm 以上で静注の β 遮断薬を使用する」など)。心拍数 60bpm 以下で撮影することにより、有意に線量低減と画質向上が得られたと報告されています。(Stocker TJ, et al. Radiology 2021; 9: 701-703.)

・prospective scan の活用によりさらなる被曝低減が見込めます。

・今回提出いただいた症例においては、心臓以外の部位の撮影が多く、心臓 CT 検査としては過剰な部分がある可能性はあります。

・撮影範囲を最適化することは被ばく線量の低減においては重要です。Ca スコア用の単純 CT を利用して左冠動脈主幹部の上 1cm から心尖の下 1cm まで撮影するのが基本です。
