# Supplementary material for: Impact of tailored feedback on optimization and radiation dose reduction in coronary CT angiography: a comparative survey between 2021 and 2023 in Mie prefecture
Source: Jpn J Radiol. 2025 Jul 19;43(11):1833–41. doi: 10.1007/s11604-025-01835-0 (PMC12575519; doi:10.1007/s11604-025-01835-0)
Supplement: Supplementary file 5 — Supplementary file5 (PDF 629 KB) [file 11604_2025_1835_MOESM5_ESM.pdf]

Cardiac CT dose survey in Mie Prefecture (2021)

~XXX hospital~

【Figure1. Total dose-length (mGy·cm) product of cardiac CT at each site】

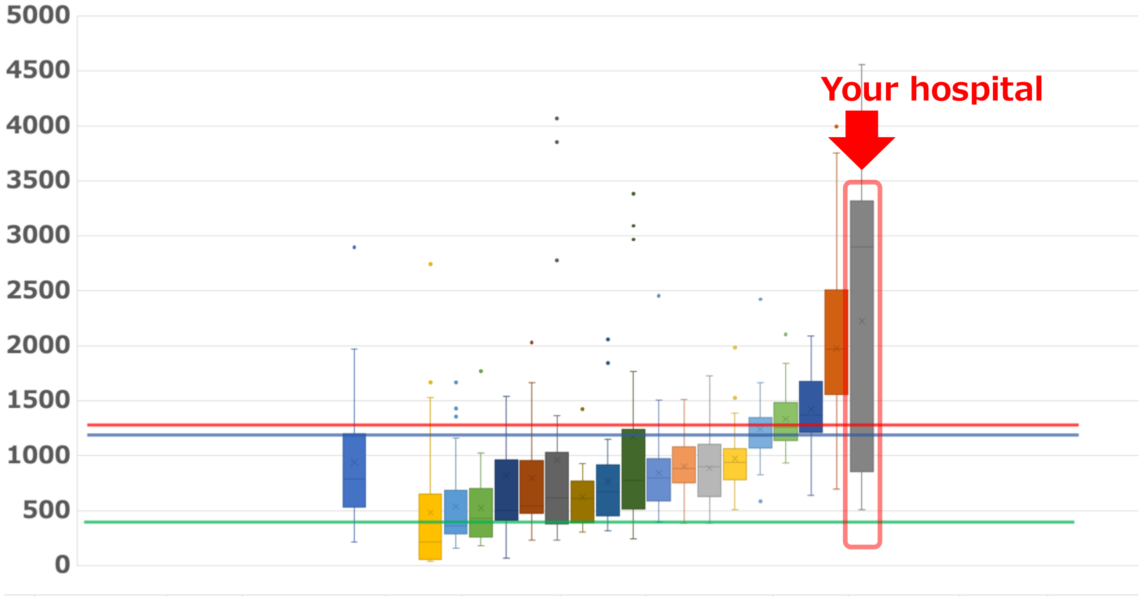

【Figure 2. Computed tomography dose index volume (mGy) of coronary CT angiography (CCTA) at each site.】

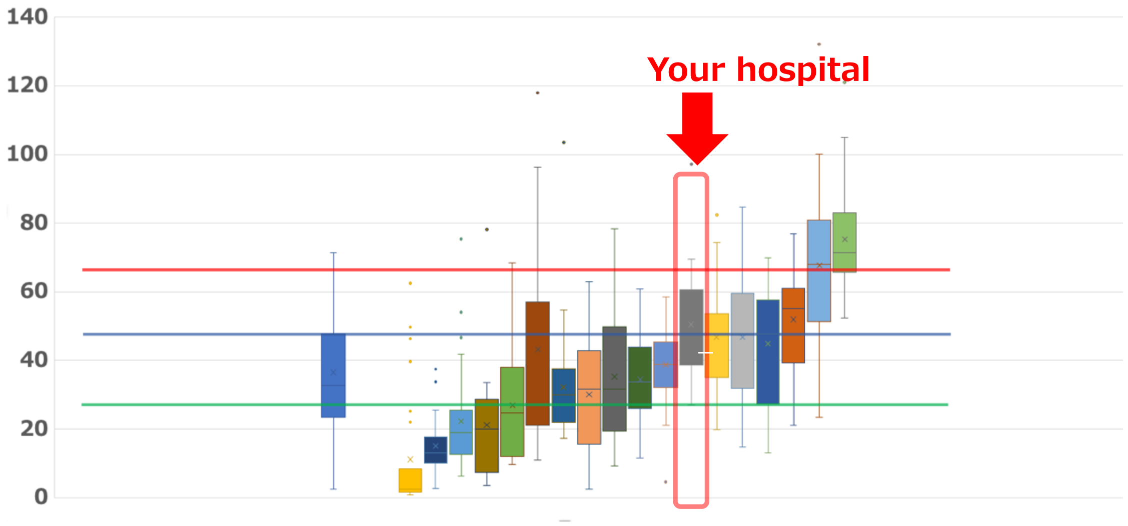

*Red line, DRL of Japan DRLs 2020; Blue line, DRL of this survey; Green line, DRL of PROTECTION VI (2017,61 facilities worldwide)

【Figure 3. Scan techniques and electrocardiogram-controlled dose modulation at each site (same order as Figure 2)】

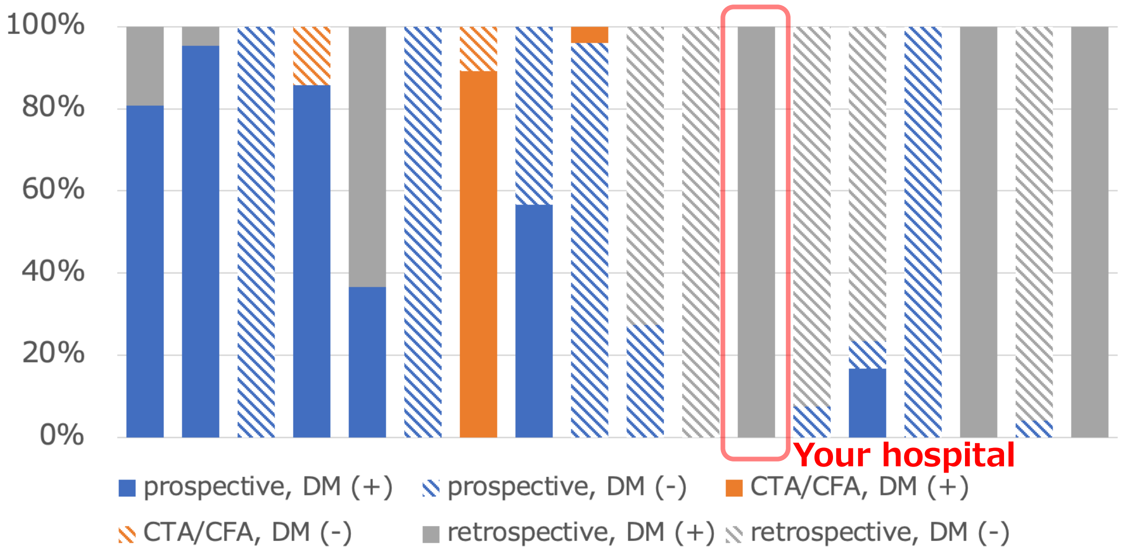

*DM: dose modulation (ECG controlled dose pulsing)

CTA/CFA: The scan mode available in Aquilion One, Canon

【Figure 4. Heart rate at the scan at each site (same order as Figure 2)】

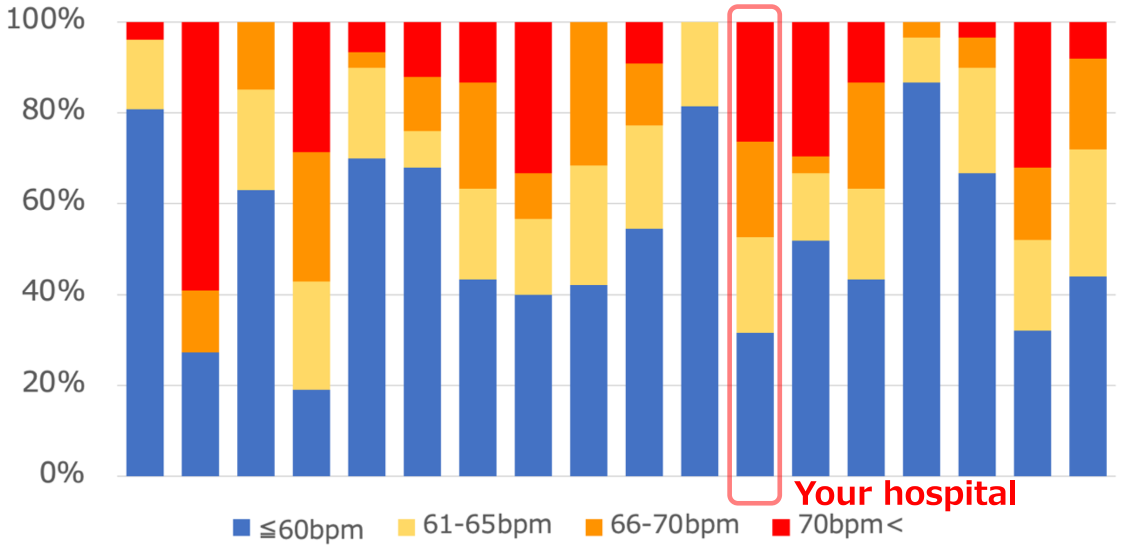

【Figure 5. Tube potential at each site (same order as Figure 2)】

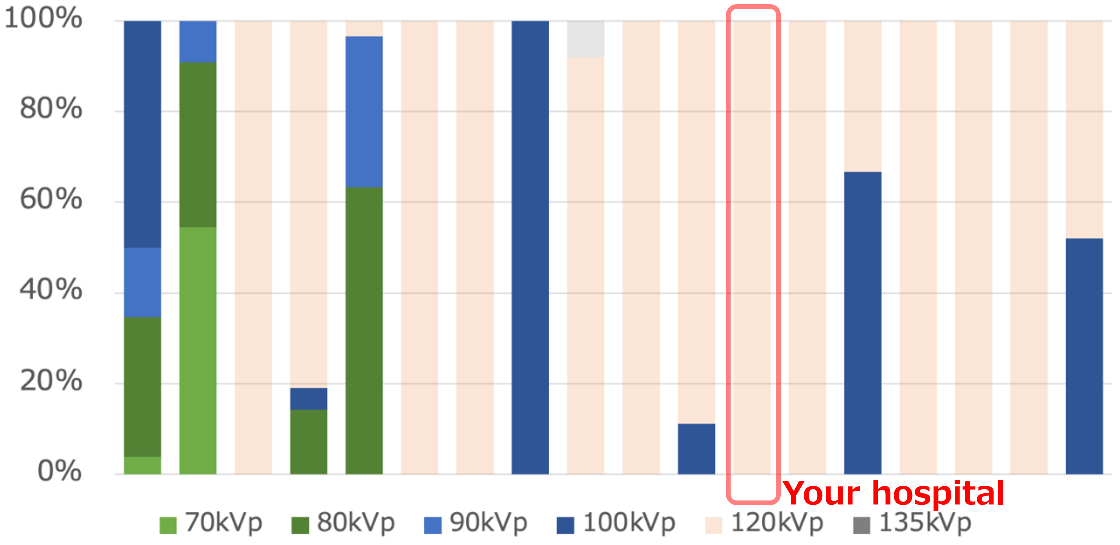

【Figure 6. Factors related to radiation exposure of CCTA】

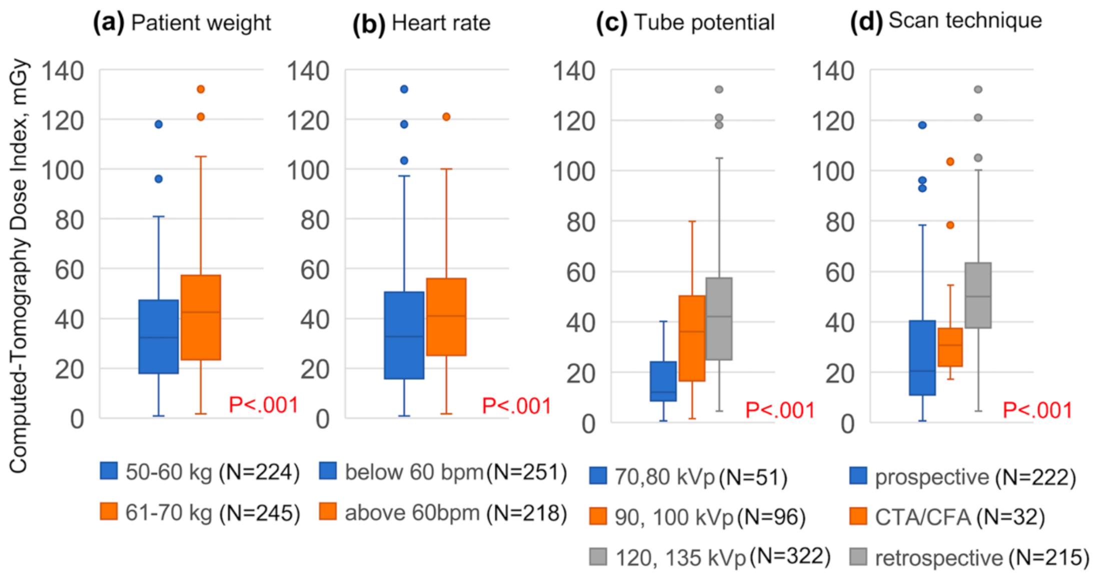

【The current status of cardiac CT examinations at your hospital compared to other facilities in the prefecture】

・The radiation dose for CCTA is comparable to the Japan DRLs 2020, but is slightly higher within the prefecture and notably higher than in global benchmark (PROTECTION VI).
・The overall radiation dose for cardiac CT examinations is higher than that observed in other facilities in the prefecture and the Japan DRLs 2020, suggesting that additional imaging of extra-cardiac regions may be excessive as cardiac CT examinations.
・More aggressive heart rate control may be feasible.
・Low tube potential imaging has not been employed.
・ECG-triggered prospective scanning has not been utilized.

【For further reduction in radiation exposure】

・Utilizing lower tube potential imaging is expected to reduce radiation exposure further. According to the SCCT guidelines, tube potential of 100 kVp is recommended for patients weighing less than 100 kg or with a BMI under 30, and tube potential of 100 kVp or 80 kVp is advised for children and smaller adults (Abbara S, et al. J Cardiovasc Comput Tomogr 2016; 10: 435-449).
・Although your facility already has your own heart rate control protocol, it may be worth considering more aggressive heart rate control (e.g. administering intravenous beta-blockers when heart rate exceeds 60 bpm). Previous study reported that CCTA imaging at a heart rate below 60 bpm can significantly reduce radiation dose and improve image quality (Stocker TJ, et al. Radiology 2021; 9: 701-703).
・Further dose reduction can also be achieved by utilizing ECG-triggered prospective scanning for the appropriate patients.
・In the cases submitted for this survey, there was a high proportion of imaging of extra-cardiac regions, which may be excessive for cardiac CT examination.
・Optimizing the scanning range, if not already done, is important for reducing radiation dose. Based on the non-contrast CT scan for calcium scoring, CCTA imaging should extend from 1 cm above the left main coronary artery to 1 cm below the cardiac apex.
